# Resonant inelastic tunneling using multiple metallic quantum wells

**DOI:** 10.1515/nanoph-2023-0231

**Published:** 2023-06-21

**Authors:** Yiyun Zhang, Dominic Lepage, Yiming Feng, Sihan Zhao, Hongsheng Chen, Haoliang Qian

**Affiliations:** Interdisciplinary Center for Quantum Information, State Key Laboratory of Extreme Photonics and Instrumentation, ZJU-Hangzhou Global Scientific and Technological Innovation Center, Zhejiang University, Hangzhou 310027, China; International Joint Innovation Center, Key Lab. of Advanced Micro/Nano Electronic Devices & Smart Systems of Zhejiang, The Electromagnetics Academy at Zhejiang University, Zhejiang University, Haining 314400, China; Jinhua Institute of Zhejiang University, Zhejiang University, Jinhua 321099, China; Institut Quantique, Université de Sherbrooke, 2500 Boulevard de l’Université, Sherbrooke, Québec J1K 2R1, Canada; Interdisciplinary Center for Quantum Information, State Key Laboratory of Silicon Materials, and Zhejiang Province Key Laboratory of Quantum Technology and Device, Department of Physics, Zhejiang University, Hangzhou 310058, China

**Keywords:** inelastic electron tunneling, internal quantum efficiency, metallic quantum wells, photon-emission power

## Abstract

Tunnel nanojunctions based on inelastic electron tunneling (IET) have been heralded as a breakthrough for ultra-fast integrated light sources. However, the majority of electrons tend to tunnel through a junction elastically, resulting in weak photon-emission power and limited efficiency, which have hindered their practical applications to date. Resonant tunneling has been proposed as a way to alleviate this limitation, but photon-emissions under resonant tunneling conditions have remained unsatisfactory for practical IET-based light sources due to the inherent contradiction between high photon-emission efficiency and power. In this work, we introduce a novel approach that leverages much stronger resonant tunneling enhancement achieved by multiple metallic quantum wells, which has enabled the internal quantum efficiency to reach ∼1 and photon-emission power to reach ∼0.8 µW/µm^2^. Furthermore, this method is applicable with different electronic lifetimes ranging from 10 fs to 100 fs simultaneously, bringing practical implementation of IET-based sources one step closer to reality.

## 1 Introduction

As the essential components of optoelectronic devices, the quest for an ideal photon source that can emit photons with ultra-fast speed, high efficiency, and sufficient power in miniaturized scale is a critical research focus [1–3]. Among the various light sources, the tunnel junctions based on inelastic electron tunneling (IET) events have gained extensive attention for their unique potential to provide ultra-compact sources with unprecedented photon modulation speed exceeding THz [4–7]. This fast photon-emission speed is attributed to the extremely short tunneling time (<10 fs) [8–10]. However, the low photon-emission efficiency (internal quantum efficiency, IQE <10 %) and radiation power (*P*_*r*_ ∼ pW/µm^2^) have severely restricted their practical applications to date. Therefore, comprehensive investigation of the fundamental mechanisms to improve the IQE and *P*_*r*_, and the interrelation between the IQE and *P*_*r*_ is imperative.

**Figure 1:**
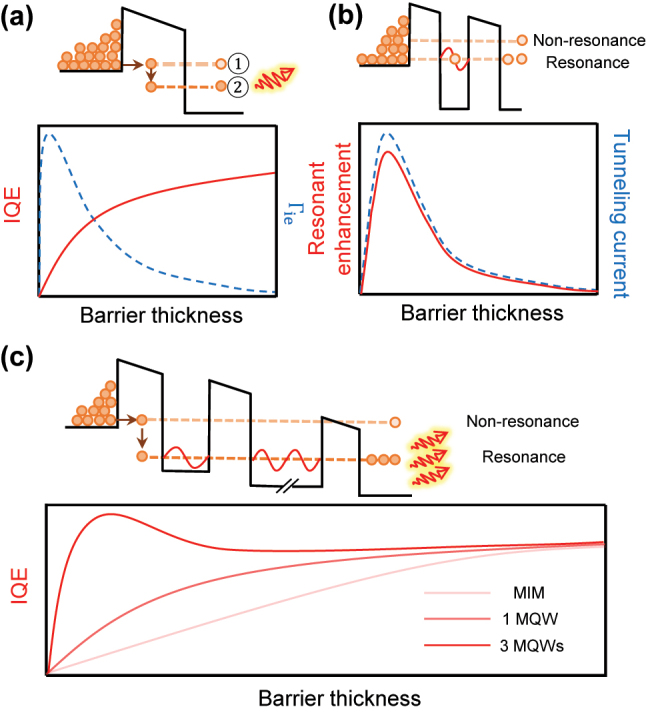
The schematic diagram of MIM, single MQW and mMQW systems. (a) The structure of classical tunnel-junctions based sources and corresponding potential energy diagram with biased field. Process (1) represents the ET events and process (2) represents the IET events. One can see clearly the opposite trend of IQE and Γ_*ie*_, indicating the contradiction between high-IQE and large-*P*_*r*_. (b) The resonance-built system formed by single MQW. By bridging one resonant tunneling channel, electrons will have higher probability across the barrier. Note that the resonant electron tunneling rate increases faster than the non-resonant electron tunneling rate. (c) The RIET-built light generation unit. By taking advantage of RIET enhancement factor utilizing 3-MWQs system, simultaneous improvement of high-IQE and large-*P*_*r*_ is possible without the unessential restrictions. Note that when the barrier thickness approaches zero, the tunneling events would become negligible.

Figure 1(a) depicts a typical metal–insulator–metal (MIM) tunnel junctions with a biased field. The quantum tunneling mechanism enables electrons to tunnel across barriers elastically or inelastically, forming a tunneling current, even if their energy is lower than the potential energy of the barriers. Elastic tunneling (ET) means electrons have no energy loss during the tunneling process (as shown in the process 1). In contrast, inelastic tunneling events (as depicted in process 2) involve electrons losing energy while tunneling, leading to photon emission [4, 11, 12]. This photon generation process can be described as a two-step process [13]: First, surface plasmons are excited by the inelastic tunneling electrons. The ratio between inelastic (Γ_*ie*_) and elastic (Γ_*e*_) tunneling rate is defined as $IQE=\frac{\Gamma_{ie}}{\Gamma_{e}+\Gamma_{ie}}$ and the radiation power *P*_*r*_ is proportional to Γ_*ie*_. Second, these plasmons may either decay radiatively in the form of photons or non-radiatively through electron-electron or electron-phonon interaction, which determines the radiation efficiency (RE). Optimizing the RE can be achieved through specifically designed structures of optical antennas; therefore, it is not the main focus of this work [14, 15]. The principle of enhancing photon-emission efficiency and power is to increase the amplitude of $\frac{\Gamma_{ie}}{\Gamma_{e}+\Gamma_{ie}}$ and Γ_*ie*_ simultaneously. Notably, contrary to most common light sources, increased Γ_*ie*_ (or emission power *P*_*r*_) does not lead to an increase in IQE. As shown in Figure 1(a), the increased Γ_*ie*_ accompanies by a decreased IQE because Γ_*e*_ can grow faster than Γ_*ie*_ from a tunneling-mechanism point of view (explained in the next section). Thus, the contradiction between high-IQE and large-*P*_*r*_ poses a significant challenge in the practical design.

Hence, a crucial aspect for the realization of optimal IET light sources is to engineer a structure that can facilitate a more rapid increase of Γ_*ie*_ as compared to the Γ_*e*_. A commonly adopted approach is to improve the optical properties of the structure by enhancing the local density of optical states (LDOS) [13, 14, 16, 17]. The enhancement of LDOS enables larger Γ_*ie*_ with minimal impacts on Γ_*e*_, leading to simultaneous growth of IQE and *P*_*r*_. However, this method will break down when the electric-field intensity is strong enough [18], limiting the maximum achievable IQE (<50 %) [17]. Moreover, the LDOS-enhancement is highly dependent on the design of intricate nanostructures, requiring complex fabrication processes. Therefore, an inevitable strategy is to engineer the tunneling rate from an additional point of view: The electrical properties through the resonance effect. When the energy of tunneling electrons matches one of the discrete energy levels formed by a quantum well (QW), the tunneling transmission coefficient can be greatly enhanced via resonant electron tunneling. More importantly, this approach demonstrates the faster increase of resonant electron tunneling rate (Γ_res_) in comparison to the non-resonant electron tunneling rate (Γ_non-res_). Consequently, the resonant enhancement ( $\frac{\Gamma_{\text{res}}}{\Gamma_{\text{non }-\text{ res}}}\left.\right)$ displays the same trend with the tunneling current, as depicted in Figure 1(b). As a result, the integration of a QW to introduce the resonant enhancement factor in IET events, specifically through the creation of a resonant IET (RIET) channel and the suppression of ET events (see details in Supplementary Material Section 1), has the potential to enhance the IQE efficiently and overcome the current contradiction between high-IQE and large-*P*_*r*_, as demonstrated by Figure 1(c). By doing so, the electron is more likely to travel through the RIET channel, leading to the enhancement of IQE. This approach is considered superior to other methods, such as the local density of states (LDOS)-enhancement method, as there is no upper limit to IQE enhancement [13, 19, 20]. Note that, if the resonant enhancement effect on the inelastic electron tunneling (IET) growth rate is insufficient, as observed in the single MQW system, the contradiction between high IQE and large *P*_*r*_ can only be partially alleviated (the lighter red curve in Figure 1(c)).

In this work, we have addressed the challenge of improving RIET enhancement by exploring the multiple metallic quantum wells (mMQWs) structures. More specifically, our study demonstrates that the tunneling transmittance in 3-MQWs resonance case is 6 orders of magnitude larger than the cases in non-resonant tunneling condition. By utilizing 3-MQWs resonance system in the tunnel junctions, we resolve the inconsistency between high-IQE and large-*P*_*r*_ (the darkest red cure in the Figure 1(c)), realizing the IQE ∼ 1 and *P*_*r*_ ∼ 0.8 µW/µm^2^ in the best cases, with an electron lifetime ranging from 10 fs to 100 fs. The proposed mMQWs-based approach offers a promising pathway to overcome the limitations of traditional IET-based photon sources and provides a viable platform for the development of ultra-fast and high-efficiency optoelectronic devices.

## 2 Results and discussion

### 2.1 The mismatch between high-IQE and large-*P*_*r*_ in MIM tunneling junctions and single MQW system

**Figure 2:**
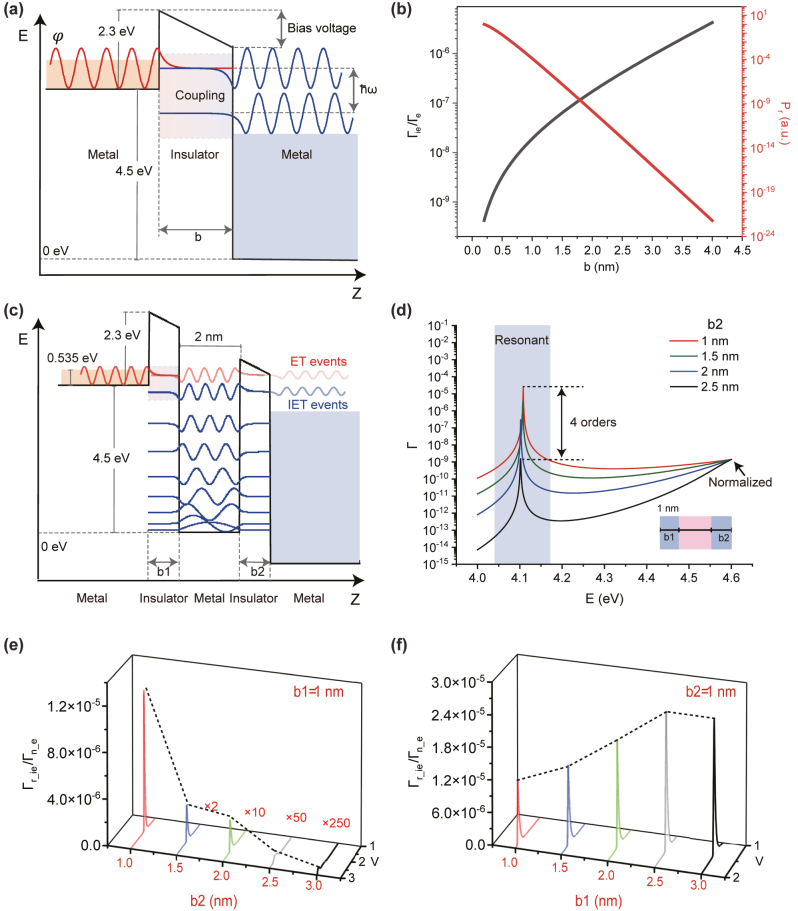
The features of IET rate with resonant enhancement brought from single MQW. (a) The potential energy and corresponding wavefunctions diagram of MIM tunneling junctions. (b) The $\frac{\Gamma_{ie}}{\Gamma_{e}}$ (black line) and normalized *P*_*r*_ (red line) distribution with barrier thickness which presents completely conversed trends with respect to barrier thickness. (c) The potential energy and wavefunctions of single MQW with RIET establishment. (d) The *E*Γ curves under different barrier thickness cases. The obvious peaks indicate the establishment of resonant tunneling. (e–f) The variation of $\frac{\Gamma_{r\text{\_}ie}}{\Gamma_{n\text{\_}e}}$ with total bias voltage (*V*) drop across the tunnel junctions. Considering that two potential barriers play distinguishable roles in the electron tunneling process, we analyze the variation of $\frac{\Gamma_{r\text{\_}ie}}{\Gamma_{n\text{\_}e}}\left(b_{1},b_{2}\right)$ with each barrier thickness *b*_1_ (e) and *b*_2_ (f), respectively. One can note that $\frac{\Gamma_{r\text{\_}ie}}{\Gamma_{n\text{\_}e}}\left(V\right)$ curves form the obvious peaks, indicating the realization of RIET-enhanced IQE when precise resonant condition of *V* is achieved. In addition, negative-slope of $\frac{\Gamma_{r\text{\_}ie}}{\Gamma_{n\text{\_}e}}\left(b_{2}\right)$ does not mean that *b*_2_ = 0 nm is the best case. Because the establishment of resonant cavity formed by double barriers is the premise of this numerical simulation [23, 24].

In the framework of the transfer-Hamiltonian formalism [21], both the Γ_*e*_ and Γ_*ie*_ can be introduced via perturbation theory and Fermi’s golden rule. Then, the relationship between IQE and *P*_*r*_ can be investigated from the simplest case: MIM tunnel junction. Here, we choose TiN as the bottom electrode/metallic layer of MQW while Al_2_O_3_ served as the insulating layer based on the lattice matching between TiN and Al_2_O_3_, which aids in the growth of single crystal Al_2_O_3_ and avoid leakage current caused by dielectric defect states. In addition, ITO is chosen as the top electrode due to its high transparency and efficient photon radiation properties. Figure 2(a) shows the potential energy of the typical MIM tunnel junction and corresponding wave functions of the left/right electrodes (*φ*_*ν*/*μ*_). In the barrier regime with thickness *b*, the *φ*_*ν*/*μ*_ decay exponentially with distance from the respective electrode, which are described as *φ*_*ν*_(*z*) = *φ*_*ν*_ *e* ^−*Kz*^ and $\varphi_{\mu }\left(z\right)=\varphi_{\mu }e^{+Kz}$ (*K* is the decay constant). The process of IET involving electron energy transitions is achieved through the coupling of electron wave functions. On the other hand, the elastic tunneling (ET) process is a direct tunneling phenomenon, as described in Supplementary Material Section 2. Due to their distinct mechanisms, the tunneling rates of ET and IET exhibit different trends with respect to tunneling distance, as elucidated in Supplementary Material Section 3. The Γ_*e*_ and Γ_*ie*_ is determined by the formulas as:

(1) $$\begin{array}{c} \Gamma_{e}=\frac{\pi \hbar^{3}}{2m^{2}}\int {\left|\left(\varphi_{\nu }\frac{d\varphi_{\mu }^{*}}{dz}-\varphi_{\mu }^{*}\frac{d\varphi_{\nu }}{dz}\right){|}_{z=z_{0}}\right|}^{2}\rho_{\mu }\left(E\right)\rho_{\nu }\left(E\right)dE, \end{array}$$

(2) $$\begin{array}{ll} \Gamma_{ie} & =\frac{2\pi \hbar e^{2}}{m^{2}}\rho \left(\omega \right)\int {\left|\int_{0}^{b}\varphi_{\mu }^{*}\left(E-\hbar \omega \right)\frac{d\varphi_{\nu }}{dz}dz\right|}^{2}\rho_{\mu }\left(E\right)\\ & \;\times \rho_{\nu }\left(E-\hbar \omega \right)d\left(E-\hbar \omega \right). \end{array}$$

Here, *E* is the incident energy of electron, *ρ*_*ν*/*μ*_ are the electronic density of states for the left/right electrons, *Z*_0_is an arbitrary point in the barrier region, $\rho \left(\omega \right)$ is defined as LDOS, and *ω* is the frequency of emitted photons. Then the *P*_*r*_ and IQE can be derived correspondingly. For the *P*_*r*_, it can be retrieved by $P_{r}\propto \Gamma_{ie}\propto {\left|be^{-Kb}\right|}^{2}$ as shown in the Figure 2(b) (red curve), which is consistent with common sense: As the barrier thickness broadens, fewer electrons can tunnel across. Thus, the radiated photon flux converted from the tunneling electrons is decreased. In regards to the IQE $=\frac{\Gamma_{ie}}{\Gamma_{ie}+\Gamma_{e}}=\frac{1}{1+\frac{1}{\Gamma_{ie}/\Gamma_{e}}}$ , the $\frac{\Gamma_{ie}}{\Gamma_{e}}$ term is proportional to IQE and shows the exact ratio between Γ_*ie*_ and Γ_*e*_ more precisely than the $\frac{\Gamma_{ie}}{\Gamma_{ie}+\Gamma_{e}}$ term. Therefore, we prefer the $\frac{\Gamma_{ie}}{\Gamma_{e}}$ term to indicate IQE. The $\frac{\Gamma_{ie}}{\Gamma_{e}}$ term can be calculated by $\frac{\Gamma_{ie}}{\Gamma_{e}}\propto \frac{{\left|be^{-Kb}\right|}^{2}}{{\left|e^{-Kb}\right|}^{2}}=b^{2}$ as the black curve shown in the Figure 2(b).

Based on the derived formula of *P*_*r*_ and $\frac{\Gamma_{ie}}{\Gamma_{e}}$ : $P_{r}\propto {\left|be^{-Kb}\right|}^{2}$ and $\frac{\Gamma_{ie}}{\Gamma_{e}}\propto b^{2}$ , we reach two conclusions: (1) *P*_*r*_ and $\frac{\Gamma_{ie}}{\Gamma_{e}}$ both are highly related to the barrier thickness *b*. Therefore, the relationship between *P*_*r*_ and $\frac{\Gamma_{ie}}{\Gamma_{e}}$ can be attained through *b*. (2) *P*_*r*_(*b*) and $\frac{\Gamma_{ie}}{\Gamma_{e}}\left(b\right)$ shows diametrically opposed trends. As shown in Figure 2(b), $P_{r}\left(b\right)$ presents a negative slope while $\frac{\Gamma_{ie}}{\Gamma_{e}}\left(b\right)$ has a positive slope. This highlights the contradiction between high-IQE and large-*P*_*r*_. Therefore, converting the slope of $\frac{\Gamma_{ie}}{\Gamma_{e}}\left(b\right)$ to be negative, to follow the same trend as $P_{r}\left(b\right)$ , would be a fundamental mechanism to eliminate the mismatch between high-IQE and large-*P*_*r*_.

A more sophisticated tunnel junction system is proposed to increase the IQE by engineering the electrical properties using RIET effect. In such RIET-built system, the potential energy and confined wave function of a double barrier structure with a QW placed between the barriers is shown in Figure 2(c). Note that, regarding the working wavelengths of IET-sources in visible and near-infrared regimes, metallic quantum wells (MQWs) are commonly utilized because they have deeper potential well and support emitting higher-energy photon compared with the semiconductor quantum wells. When the incident energy of electron matches the discrete energy level formed by the MQW, a resonant tunneling phenomenon occurs. The resonant tunneling induces a large transmission coefficient attributed to the constructive interference between the wavefunctions transmitted and reflected from the barriers [22]. Therefore, adding resonant tunneling channels to IET events while non-resonant paths for the ET transmission is an effective strategy to enhance the IQE [13]. Considering the RIET system built by a MIMIM tunneling structure with well width of *a* and the surrounding barriers width as *b*_1_ and *b*_2_, the function of resonant IET rate(Γ_*r*_*ie*_) and non-resonant ET rate (Γ_*n*_*e*_) are found to be (see details in Supplementary Material Section 4):

(3) $$\begin{array}{c} \Gamma_{n\text{\_}e}\left(b_{1},b_{2}\right)=\int \Gamma \left(E,b_{1},b_{2}\right)\rho_{\mu }\left(E\right)\rho_{\nu }\left(E\right)dE, \end{array}$$

(4) $$\begin{array}{ll} \Gamma_{r\text{\_}ie}\left(b_{1},b_{2}\right) & =\frac{\Gamma_{ie}}{\Gamma_{e}}\left(b_{1}\right)\int \Gamma \left(E-\hbar \omega ,b_{1},b_{2}\right)\rho_{\mu }\left(E\right)\\ & \;\times \rho_{\nu }\left(E-\hbar \omega \right)dE\cdot \end{array}$$

Here, Γ is original tunneling transmission coefficients which is calculated by piece-wise linear approximation method. $\frac{\Gamma_{ie}}{\Gamma_{e}}\left(b_{1}\right)$ term is regarded as IET-coupling efficiency which is obtained from Eqs. (1)–(2). Note that, the first barrier (*b*_1_) determines the IET-coupling efficiency while the second barrier has trivial effect on this, because the probability of IET events happened in the second potential is negligible without any IET enhancement.

Then the $\frac{\Gamma_{r\text{\_}ie}}{\Gamma_{n\text{\_}e}}\left(b_{1},b_{2}\right)$ can be obtained approximately as $\frac{\Gamma_{r\text{\_}ie}}{\Gamma_{n\text{\_}e}}\left(b_{1},b_{2}\right)\propto \frac{\frac{\Gamma_{ie}}{\Gamma_{e}}\left(b_{1}\right)\int \Gamma \left(E-\hbar \omega ,b_{1},b_{2}\right)dE}{\int \Gamma \left(E,b_{1},b_{2}\right)dE}$ , which is positively related to resonant enhancement effects ( $\frac{\int \Gamma \left(E-\hbar \omega ,b_{1},b_{2}\right)dE}{\int \Gamma \left(E,b_{1},b_{2}\right)dE}$ ). In order to investigate the characteristics of resonant enhancement, the numerical simulations of *E*Γ curves with fixed first barrier thickness (*b*_1_) as 1 nm and varied second barrier thickness (*b*_2_) as 1 nm, 1.5 nm, 2 nm, and 2.5 nm are carried out (as shown in the Figure 2(d)). Here, we normalize the tunneling transmission coefficient Γ to the *b*_2_ = 1 nm case by keeping the same amplitude at 4.6 eV energy level. Firstly, one can see the resonant tunneling transmittance is ∼10^4^ times higher than the non-resonant tunneling transmittance. More importantly, the increasing trend of normalized Γ in resonant energy range indicates the faster increase of resonant tunneling transmittance compared with non-resonant tunneling transmittance. Therefore, as expected, $\frac{\Gamma_{r\text{\_}ie}}{\Gamma_{n\text{\_}e}}\left(b_{2}\right){|}_{b_{1}=1\;nm}$ shows an increasing trend (black dashed line) with *b*_2_ decreased as shown in the Figure 2(e). Such properties show an exact opposite trend to the classical MIM tunnel junctions system (black curve in the Figure 2(b)) and provide the desired trends for optimal light sources. Although simultaneous increases in photon emission efficiency and power have been achieved within a certain range, the IQE remains below 10^−4^ even the *b*_2_ has been reduced to 1 nm. This limitation stems from inadequate resonant enhancement which fails to compensate for the weak IET-coupling efficiency ( $\frac{\Gamma_{\text{r}\text{\_}\text{ie}}}{\Gamma_{\text{n}\text{\_}\text{e}}}\left(b_{1}\right)$ ) to enhance the inelastic tunneling rate to a level comparable to the elastic tunneling rate. Then in order to tune the IET-coupling efficiency that is determined by the first barrier, the variation of $\frac{\Gamma_{r\text{\_}ie}}{\Gamma_{n\text{\_}e}}\left(b_{1}\right){|}_{b_{2}=1\;nm}$ is represented in Figure 2(f). The positive slope of $\frac{\Gamma_{r\text{\_}ie}}{\Gamma_{n\text{\_}e}}\left(b_{1}\right){|}_{b_{2}=1\;nm}$ means that to increase the IET-coupling efficiency, *b*_1_ need to be larger, resulting in a decrease of total *P*_*r*_. Therefore, the desired increase for both the IQE and *P*_*r*_ is limited again, even adding the tunable thickness of *b*_2_ as one more degree of freedom. Nevertheless, the positive slope of $\frac{\Gamma_{r\text{\_}ie}}{\Gamma_{n\text{\_}e}}\left(b_{1}\right){|}_{b_{2}=1\;nm}$ curve is much gentler compared with the typical MIM tunnel junction case, which indicates the potential contribution from resonant enhancement brought from RIET channels.

### 2.2 The elimination of contradictions in mMQWs system

**Figure 3:**
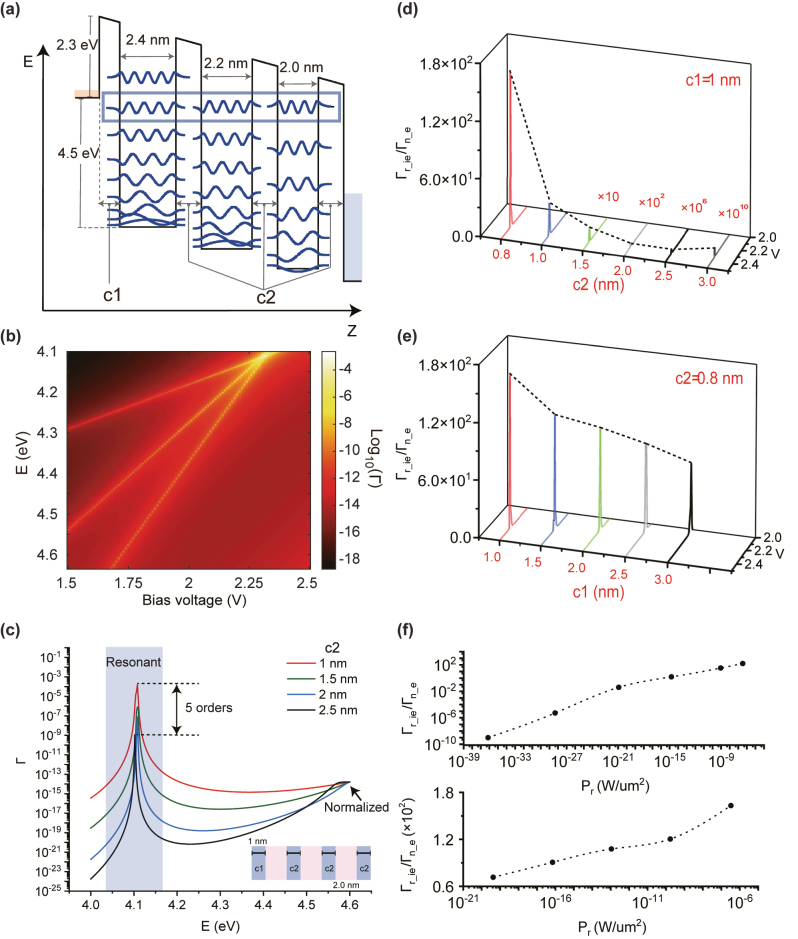
The breakdown of contraction between high-IQE and large *P*_*r*_ by mMQW systems. (a) The potential energy and corresponding wavefunctions of mMQWs system. (b) The *EV*Γ map of the engineered mMQWs system. (c) The *E*Γ curves under resonant voltages with different *c*_2_. It is worth to note that, by considering the practical fabrication with certain thickness variation, such RIET enhancement from mMQWs may give a relatively lower value (see detailed discussion in Supplementary Material Section 10). (d–e) The variation of $\frac{\Gamma_{r\text{\_}ie}}{\Gamma_{n\text{\_}e}}$ with *V* at several different barrier thickness (*c*_1_ and *c*_2_) cases. (f) The corresponding relationship between $\frac{\Gamma_{r\text{\_}ie}}{\Gamma_{n\text{\_}e}}$ and *P*_*r*_ for (d) and (e), respectively. Here, considering the typical electron dephasing time as 10–100 fs [25], we select the best case as 100 fs for the representative, and other electron lifetime cases will be discussed in the next section.

A straightforward next step is to further improve the effect of resonant enhancement and reverse the trend of $\frac{\Gamma_{r\text{\_}ie}}{\Gamma_{n\text{\_}e}}\left(b_{1}\right)$ . Electron resonant tunneling realized by MQW structure is analogous to an optical resonant case: inspired by the high-Q value realized by the multiple optical resonant cavities, a cascading MQWs structure is proposed to increase the RIET-enhancement effect. The proposed ITO/Al_2_O_3_/TiN/Al_2_O_3_/TiN/Al_2_O_3_/TiN mMQWs structure is illustrated in Figure 3(a). By engineering the width of each quantum well and barrier, the precise alignment of three IET-resonant energy levels at one specific voltage can be realized. As shown in the *EV*Γ map of the mMQWs structures (Figure 3(b)), the three bright bands correspond to the variation of discrete energy levels formed by each quantum well. The intersection point indicating that all of them are aligned well, achieving the 3-MQWs multiple resonant tunneling. Similarly, because the first barrier determines the IET-coupling efficiency, we will analyze such multiple RIET enhancement characteristics from two aspects, including the dependence on the first barrier thickness *c*_1_ and the total thickness of the remaining barriers 3 × *c*_2_ (as indicated in the Figure 3(a)). Figure 3(c) shows the *E*Γ curves under resonant voltages with fixed *c*_1_ as 1 nm and varied *c*_2_ as 1 nm, 1.5 nm, 2 nm, and 2.5 nm. The transmittance in such 3-MQWs resonant tunneling case is 10^10^ times higher than the transmittance in non-resonant tunneling case. Compared with ∼10^4^-fold RIET-resonant enhancement from single MQW tunneling system, the stronger resonant enhancement (10^10^) further prove the effectiveness of the mMQWs structure. Additionally, the mMQWs system exhibits higher Q-factor values compared to the single MQW system, helping to enhance IQE more effectively (see details in Supplementary Material Section 5). Accompanied with such stronger RIET-enhancement effect, the increasing amplitude of resonant tunneling transmittance (with barrier thickness decreased from 2.5 nm to 1 nm) becomes tenfold larger than the single MQW case. Figure 3(d) shows the $\frac{\Gamma_{r\text{\_}ie}}{\Gamma_{n\text{\_}e}}\left(c_{2}\right){|}_{c_{1}=1\;nm}$ diagram. As expected, $\frac{\Gamma_{r\text{\_}ie}}{\Gamma_{n\text{\_}e}}\left(c_{2}\right){|}_{c_{1}=1\;nm}$ curves show the steeper negative slope than the single MQW system. More importantly, the slope of the $\frac{\Gamma_{r\text{\_}ie}}{\Gamma_{n\text{\_}e}}\left(c_{1}\right){|}_{c_{2}=0.8\;nm}$ is now changed to be negative (as shown in Figure 3(e)) contributed by enhanced growing rate of resonant tunneling transmittance. Finally, the unified increase of IQE and *P*_*r*_ is successfully realized. Here, *c*_1_ = 0 nm or *c*_2_ = 0 nm does not mean the best cases. Because the wavefunction would tunnel through the barrier and strong coupling between wavefunctions will occur when the barrier thickness is small enough, resulting the splitting and mismatch of resonant energy levels, as well as the breakdown of built RIET enhancement (Supplementary Material Section 6). Last but not least, in order to evaluate the value of *P*_*r*_ realistically, a numerical simulation of the IET current is carried out (Supplementary Material Section 7). Figure 3(f) represents the relationship between *P*_*r*_ and $\frac{\Gamma_{r\text{\_}ie}}{\Gamma_{n\text{\_}e}}$ corresponding to the cases described in Figure 3(d and e), respectively, indicating the current mMQWs structure could realize an IQE ∼ 1 and *P*_*r*_ ∼ 0.8 µW/µm^2^ in the best cases.

In addition, to gain a more intuitive understanding of how mMQWs can simultaneously achieve high photon-emission power and efficiency, we draw an analogy between the resonant tunneling behavior of electrons and the resonant transmittance behavior of photons in a Fabry–Perot cavity. Generally, for the mMQWs system, the presence of an ultra-strong resonance indicated by a high Q-factor, provides the IQE-improvement basis and promote effective functioning of resonant enhancement realizing both high photon-emission power and efficiency simultaneously (see details in Supplementary Material Section 8). However, one should note that more metal quench and dielectric defects will be induced by adding more layers of MQW, reducing the radiation efficiency of generated photons in mMQW system. Thus, the evaluation of additional loss introduced by the metal quench is performed (see details in Supplementary Material Section 9). The absorption spectrum demonstrates a ∼15–20 % increase as the number of MQW increased from 1 to 3, which has minimal impact on the significant resonant enhancement effect around 10^10^.

### 2.3 The IQE and *P*_*r*_ with varied electron lifetime

**Figure 4:**
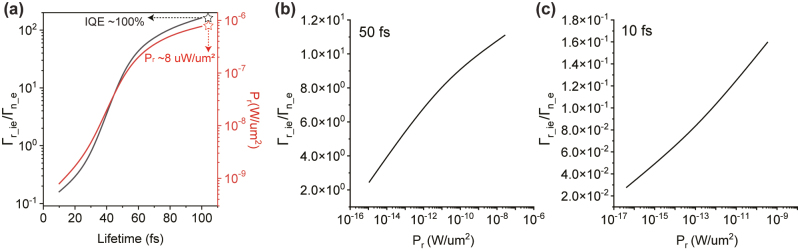
The mMQW system with varied electron lifetime. (a) The variation of $\frac{\Gamma_{r\text{\_}ie}}{\Gamma_{n\text{\_}e}}$ and *P*_*r*_ with different electron lifetime. The detail information of mMQWs is represented in the inset. (b–c) The $\frac{\Gamma_{r\text{\_}ie}}{\Gamma_{n\text{\_}e}}-P_{r}$ curves for different lifetime cases (50 fs for (b) and 10 fs for (c)). Note that, the *P*_*r*_ is adjusted by varied *c*_1_.

In regarding to practically fabricating the cascaded MQWs system, achieving precise matching of three energy levels under a single bias voltage necessitates high precision in the film processing. Thus, we further discuss the actual device performance with considering the experimental aspects including the effects of thickness variation and defects. Firstly, we investigate the impact of thickness variation on key device parameters. Specifically, when the quantum well thickness deviates by 0.2 nm (approximately one atomic layer), the resonant enhancement factor is reduced from ∼10^10^ to 10^7^ due to the shift in resonant energy levels (Supplementary Material Section 10). To address this challenge, in addition to the epitaxial growth of single crystal layers, such as TiN and Al_2_O_3_ utilized in the present modeling, an alternative approach involves employing a transfer-technique that utilizes two-dimensional materials such as insulating h-BN layers and metallic films [26]. This approach holds promise in meeting the required specifications and enables the construction of a two-dimensional tunneling junction composed of multiple single-crystal monolayers bounded by van der Waals forces. Consequently, the resulting film exhibits ultra-smooth surfaces and precise control over atomic layer thickness, ensuring the desired IQE and photon-emission power [27–29]. Secondly, we investigate the potential impact of defects within the multiple metallic films constituting the device. These defects have the potential to affect the electronic lifetime, with a higher density of defects leading to a shorter electronic lifetime due to increased electron-electron and electron-phonon collisions [30, 31]. Thus, it is necessary to check whether the relationship between $\frac{\Gamma_{r\text{\_}ie}}{\Gamma_{n\text{\_}e}}$ and *P*_*r*_ is still satisfactory in such mMQWs system with different electron lifetime cases. The electron lifetime represents the possibility of the tunneling electron experiencing electron-phonon and electron-electron collisions, which break the coherence and broadens the possible energy range of tunneling electrons [32]. This energy broadening can be approximately expressed as $\Delta E=\frac{\hbar }{2\Delta \tau }$ based on the uncertainty principle. This means shorter electron lifetimes will lead to a larger uncertainty in the electron energy, weakening the strength of the resonant enhancement and amplitude of IQE/*P*_*r*_ as shown in the Figure 4(a). Here, the electronic lifetime ranges from 10 fs to 100 fs considering the typical electron dephasing time of TiN [25]. In addition, we select 10 fs and 50 fs as the representative electron lifetime for most of the plasmonic materials, respectively, and the numerical $\frac{\Gamma_{r\text{\_}ie}}{\Gamma_{n\text{\_}e}}$ -*P*_*r*_ curve simulations are carried out as shown in the Figure 4(b) and (c). The expected proportional relationship proves the effectiveness of the cascaded MQWs structure further.

## 3 Conclusions

In conclusion, we demonstrate that multiple RIET enhanced inelastic electron tunneling events have the potential of eliminating the contraSectdiction between photon-emission power and efficiency. Our proposed three cascaded metallic quantum wells (mMQWs) based tunnel junctions structure is demonstrated to amplify the effect of resonant enhancement, realizing an IQE ∼ 1 and *P*_*r*_ ∼ 0.8 µW/µm^2^ in the best cases with electron lifetime ranging from 10 fs to 100 fs. Theoretically, the performance can be further enhanced by increasing the number of quantum wells, albeit at the expense of more refined fabrication. The proposed structure formed by stacked multilayers can be fabricated straightforwardly by film deposition with high precision [33–36] or by employing transfer-techniques utilizing two-dimensional materials, making it practical for future use in high-efficiency optoelectronic devices. In additional, the maximum emission wavelength range would be tuned by the fermi energy of electrodes. By incorporating high-efficiency optical antennas, we can maximize the external quantum efficiency and enable the realization of quantum light sources in the visible/near-infrared ranges, including single-photon or entangled photons [17], including single-photon or entangled photons [37]. Thus, the implementation of strong RIET-enhanced tunneling structures could open up possibilities for ultra-fast, high-efficiency photon sources for high-performance photonic and plasmonic circuitries.

## Supplementary Material

Supplementary Material Details
