# Supplementary material for: Resonant inelastic tunneling using multiple metallic quantum wells
Source: Nanophotonics. 2023 Jun 21;12(16):3313–21. doi: 10.1515/nanoph-2023-0231 (PMC11501182; doi:10.1515/nanoph-2023-0231)
Supplement: Supplementary file 1 — Supplementary Material Details [file j_nanoph-2023-0231_suppl_001.docx]

**Supplementary Material**

**Resonant Inelastic Tunneling using Multiple Metallic Quantum Wells**

Yiyun Zhang^1,2,3^, Dominic Lepage^4^, Yiming Feng^1,2,3^, Sihan Zhao^5*^, Hongsheng Chen^1,2,3*^, Haoliang Qian^1,2,3*^

^1^Interdisciplinary Center for Quantum Information, State Key Laboratory of Extreme Photonics and Instrumentation, ZJU-Hangzhou Global Scientific and Technological Innovation Center, Zhejiang University, Hangzhou 310027, China.

^2^International Joint Innovation Center, Key Lab. Of Advanced Micro/Nano Electronic Devices & Smart Systems of Zhejiang, The Electromagnetics Academy at Zhejiang University, Zhejiang University, Haining 314400, China.

^3^Jinhua Institute of Zhejiang University, Zhejiang University, Jinhua 321099, China.

^4^Institut Quantique, Université de Sherbrooke, 2500 Boulevard de l'Université, Sherbrooke, Québec J1K 2R1, Canada.

^5^Interdisciplinary Center for Quantum Information, State Key Laboratory of Silicon Materials, and Zhejiang Province Key Laboratory of Quantum Technology and Device, Department of Physics, Zhejiang University, Hangzhou 310058, China.

**Section S1. Resonant inelastic tunneling mechanism**

As depicted in Figure S1, resonant tunneling can occur only when the energy level of the electron aligns with the resonance energy level. Consequently, it becomes possible to manipulate the distribution of discrete energy levels formed by the metallic quantum well (MQW) through adjustments in its width. By precisely matching the resonant energy level exclusively to the energy of inelastic tunneling electrons and not elastic tunneling electrons, the resonant tunneling process can effectively enhance the inelastic tunneling rate while leaving the elastic tunneling rate unaffected [1].


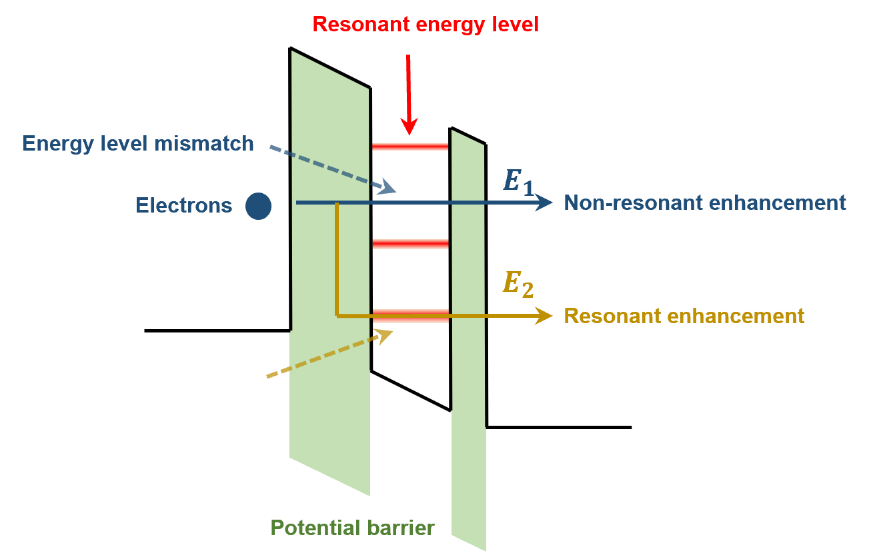


**Figure S1.** The resonant inelastic tunneling process and non-resonant elastic tunneling process.

**Section S2. Different mechanisms and trends of tunneling rates between elastic electron and inelastic electron tunneling process**

In the inelastic tunneling process, as depicted in Figure S2(a), electron transitions from state $E_{1}$ to $E_{2}$ occur through the coupling of electron wave functions. The efficiency of this coupling is determined by the local density of states (LDOS) and the inelastic tunneling rate. The vacuum LDOS can be calculated as ${(\frac{E}{\hbar})}^{2}c^{-3}\pi^{-2}$, where E represents the energy difference between $E_{2}$ and $E_{1}$, c is the speed of light in vacuum, and ℏ is the reduced Planck constant. Notably, when $E_{2}$ equals $E_{1}$ (E = 0), as illustrated in Figure S2(b), the coupling-enabled tunneling process cannot occur, leading to direct elastic tunneling without coupling.

Due to the distinct working mechanisms of elastic and inelastic tunneling, their tunneling rates exhibit different trends with respect to tunneling distance. Figure S2(c) demonstrates that the elastic tunneling rate ($\Gamma_{e}$) exponentially decreases with tunneling distance b, described as $\Gamma_{e}\propto\left| e^{-Kb} \right|^{2}$, where K represents the decay constant and b denotes the tunneling distance. On the other hand, the inelastic tunneling rate ($\Gamma_{ie}$)is derived as $\Gamma_{ie}\propto\left| {be}^{-Kb} \right|^{2}$, decaying more slowly with tunneling distance due to the influence of long-distance coupling. Consequently, the term $\frac{\Gamma_{ie}}{\Gamma_{e}}$, which affects the IQE, exhibits contrasting trends with the radiated photon flux ($\propto\Gamma_{ie}$). This stark difference underscores the inherent contradiction between photon emission efficiency and power.


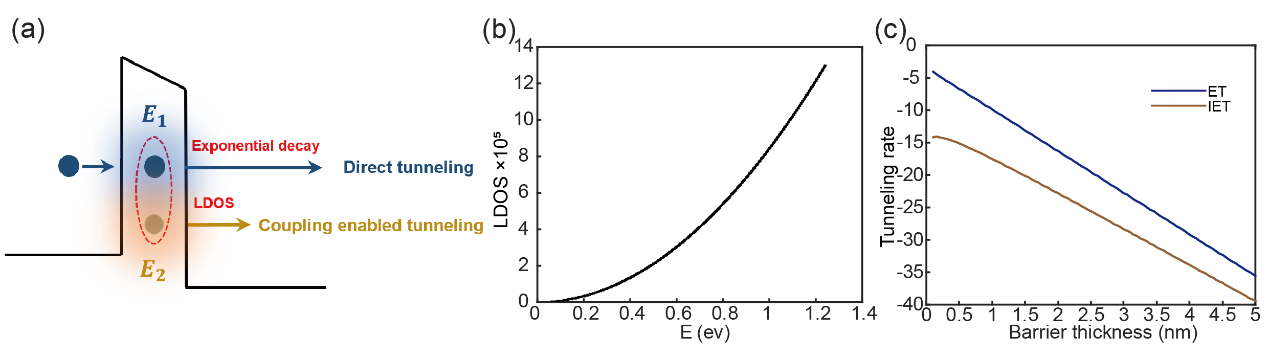


**Figure S2.** The reasons for the contradiction between photon emission efficiency and power. (a) The schematic figure of different working mechanisms of the elastic and inelastic tunneling processes. Here, the elastic tunneling is a direct tunneling behavior and inelastic tunneling is a coupling enabled tunneling behavior. (b) The calculated vacuum LDOS. (c) The tunneling rate of elastic and inelastic tunneling process varied with tunneling distance.

**Section S3. Calculation method for classical elastic and inelastic tunneling rate**

In the framework of the transfer-Hamiltonian formalism, both the $\Gamma_{e}\mathrm{and}\Gamma_{ie}$ can be introduced via perturbation theory and Fermi’s golden rule [2]. Then, $\Gamma_{e}$ reads as: $\Gamma_{e}=\frac{2\pi}{\hbar} {t_{\nu\mu}}^{2} \delta\left( E_{\mu}-E_{\nu} \right).$Here, $\hbar$ is the reduced Planck constant, $t_{\nu\mu}=\left\langle\varphi_{\nu} | \mathcal{H}_{el} | \varphi_{\mu} \right\rangle$ is the transfer matrix element for ET events representing ET-coupling efficiency and $\mathcal{H}_{el}=\Theta\left( z-z_{0} \right)\left[ U_{\nu}\left( z \right)-U_{\mu}\left( z \right) \right].$ With $U_{\nu/\mu}$ and $\varphi_{\nu/\mu}$ being the potential energies and wave functions of the left/right electrodes respectively, $Z_{0}$is an arbitrary point in the barrier region. The fully integrated $\Gamma_{e}$ is given by $\Gamma_{e}=\frac{2\pi}{\hbar}\int_{0}^{eV_{b}} \left| \Gamma\left( E \right) \right|^{2}\rho_{\mu}\left( E \right)\rho_{\nu}\left( E \right)dE.$ $\rho_{\nu/\mu}$ are the electronic densities of states for the left/right electrons and $\Gamma\left( E \right)=\frac{\hbar^{2}}{2m}{(\varphi_{\nu}\frac{d\varphi_{\mu}^{*}}{dz}-\varphi_{\mu}^{*}\frac{d\varphi_{\nu}}{dz})|}_{z=z_{0}}$.

$\Gamma_{ie}$ can be derived as $\Gamma_{ie}=\frac{2\pi}{\hbar}\sum_{l} \left| \left\langle\varphi_{\nu},\{1_{\omega_{l}}\} | \mathcal{H}_{inel} | \varphi_{\mu},\{0\} \right\rangle\right|^{2}\delta\left( \omega-\omega_{l} \right) \delta\left( E_{\mu}-E_{\nu}-\hbar\omega_{l} \right)$. $\omega$ is the angular frequency and $\hbar\omega_{l}$ represents the transition energy. $\{1_{\omega_{l}}\}$ and $\{0\}$ are one-photon state and zero-photon state, respectively. $\mathcal{H}_{inel}$ is given by the light-matter interaction Hamiltonian and simply reads as $\mathcal{H}_{inel}=-\frac{e}{m} \hat{A} \hat{P}$. $m$ and $e$ are the effective electron mass and charge. $\hat{A}$ is the vector potential operator and $\hat{P}$ is the momentum operator. The vector potential can be separated from the expectation value assuming the wave vector of the electromagnetic field is negligible compared to the electronic wave vector (dipole approximation). That is, spectral $\Gamma_{ie}$ can be simplified as: $\Gamma_{ie}=\frac{2\pi e^{2}}{\hbar m^{2}}\rho(\omega)\left| P_{\mu,\nu} \right|^{2}$. Here, $\rho\left( \omega\right)=\left| \left\langle1_{\omega_{l}} | \hat{A} | 0 \right\rangle\right|^{2}$ is defined as LDOS representing the optical properties of the tunneling system. $P_{\mu,\nu}= \left\langle\varphi_{\nu} | \hat{P} | \varphi_{\mu} \right\rangle$ is independent of the optical properties of the system and can be viewed as the source spectrum of IET. Finally, the spectral $\Gamma_{ie}$ without LDOS enhancement are given by $\frac{\pi e^{2}}{3\hbar\omega m^{2}\varepsilon_{0}}\rho_{o}\int_{h\nu}^{eV_{b}} \left| \mathcal{P}\left( E,\hbar\omega\right) \right|^{2}\rho_{\mu}\left( E-\hbar\omega\right)\rho_{\nu}\left( E \right)dE.$ Here, $\varepsilon_{0}$ is the vacuum permittivity. $\mathcal{P}\left( E,\hbar\omega\right)=-i\hbar\int_{0}^{b} \varphi_{\mu}^{*}(E-\hbar\omega)\frac{d\varphi_{\nu}}{dz}dz,$ $b$ is the thickness of barriers for MIM tunnel junctions (as shown in the Figure 2a).

**Section S4. Calculation method for non-resonant elastic and resonant inelastic tunneling rate**

For the RIET-built MQWs system, the discrete energy levels can be aligned well under the specific bias voltage, establishing the resonant channel. However, the transmission coefficient of resonant inelastic and non-resonant elastic tunneling events cannot be calculated easily based on the coupling of electron wave functions as explained in Calculation Section 1. Here, piece-wise linear approximation method provides a solid strategy to simulate the numerical elastic tunneling rate in multiple MQW system [3, 4]. It approximates real barrier with piece-wise linear segments to work out the solution of the Schrodinger equation, obtaining the elastic transmission coefficient. Therefore, the non-resonant elastic tunneling rate ($\Gamma_{n\_e}$) can be deduced as $\Gamma_{n\_e}(b_{1},\ldots, b_{i})=\int\Gamma_{non-res}(E,b_{1},\ldots, b_{i})\rho_{\mu}\left( E \right)\rho_{\nu}\left( E \right)dE$. Here, $\rho_{\nu/\mu}$ are the electronic densities of states for the left/right electrons, $E$ is the incident energy of electron, $\Gamma_{non-res}\left( E, b_{1},\ldots, b_{i} \right)$ are non-resonant tunneling transmission coefficient and $b_{1},\ldots, b_{i}$ are the thickness of each barrier, $i$ is the total number of barriers.

Regarding to the inelastic transmission coefficient, it can be divided into two parts, namely as the IET-coupling efficiency in the first barrier system and resonant tunneling rate in the whole MQW system. Such process can be explained as follows. When the electron undergoes the resonance-enhanced elastic tunneling channel, the transmission coefficients ($\Gamma_{res}$) can be calculated based on piece-wise linear approximation method. In the exactly inelastic resonant tunneling case, the wave function coupling efficiency for the inelastic transition in the first barrier leads to the tunneling transmission difference between inelastic and elastic tunneling events. Therefore, the RIET rate is calculated as $\Gamma_{r\_ie}(b_{1},\ldots, b_{i})=\frac{\Gamma_{ie}}{\Gamma_{e}}\left( b_{1} \right)\int\Gamma_{res}\left( E, b_{1},\ldots, b_{i} \right)\rho_{\mu}\left( E \right)\rho_{\nu}\left( E-\hbar\omega\right)d(E-\hbar\omega)$. Where, $\hbar\omega$ is the energy of emitted photon, $\Gamma_{res}\left( E, V,b_{1},\ldots, b_{i} \right)$ is the resonant tunneling transmission coefficient. In addition, the $\frac{\Gamma_{ie}}{\Gamma_{e}}\left( b_{1} \right)$ term is used to transfer the elastic resonant transmission coefficients to inelastic resonant transmission coefficients.

**Section S5. The enhancement benefit from 3-MQWs system**

When the number of MQWs increases to 3, forming the multiple MQWs (mMQWs) system, the resonance enhancement is significantly amplified to 10^10^, thereby providing a substantial improvement in IQE (as demonstrated in Figure S3(a)). Additionally, the mMQWs system exhibits higher Q-factor values compared to the single MQW system (Figure S3(b-c)). Only under conditions of sufficiently high Q-factor can the resonance enhancement effectively play the role of enhancing IQE, as illustrated in Figure S7.


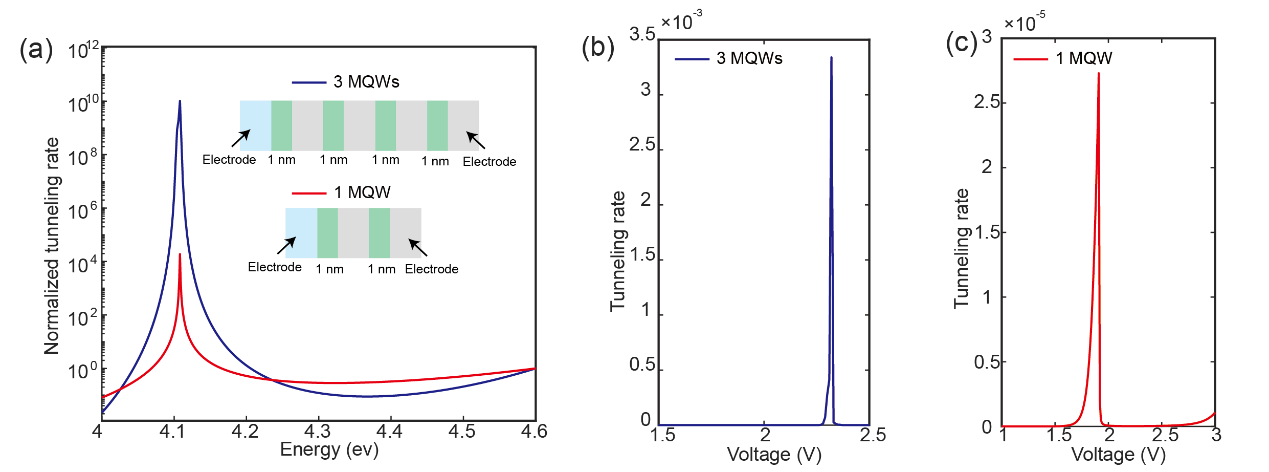


**Figure S3.** The comparison of single MQW system and mMQWs system. (a) The tunneling rate of single MQW system and mMQWs system with different electron energies. Here, we normalize the tunneling rate by keeping tunneling rate as 1 at 4.6 Ev energy level. (b-c) The tunneling rate of single MQW system (b) and mMQWs system (c) under different bias voltages. One can see that mMQWs system exhibits a significantly higher Q-factor compared to single MQW system.

**Section S6. The mismatch of resonance energy levels due to strong coupling effect of wavefunctions**

As explained in the manuscript, when the barrier thickness is thin enough to result in the strong coupling of wavefunctions, resonant energy levels will be divergent as shown in the Figure S4.


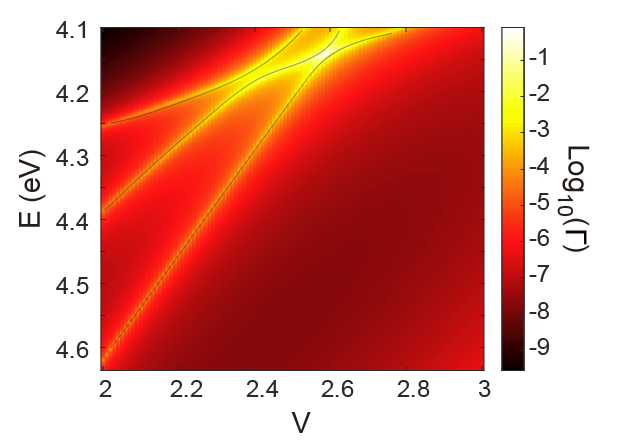
­

**Figure S4**. The $EV\Gamma$ map of mMQWs system with all the barrier thickness being reduced to 0.5 nm.

**Section S7. The calculation of tunneling current in three-dimensional devices**

Considering three-dimensional free space, the electrons can be launched in any direction with equal probability through a solid angle of $2\pi$ as show in the left-hand side of Figure S5. The current formed by tunneling electrons depends only on its wave vector along the $z$ direction ($k_{z}$). Then the problem of calculating the current is simplified to computing the number of total tunneling electrons and their velocity along $z$ direction ($v_{z}$) under the specific electron distribution. The number of total tunneling electrons can be obtained by the convolution of tunneling transmission coefficient ($\Gamma(E,V)$), free-electron density of states ($\rho(E,V)$) and energy distribution (σ($E,V$)). The $\Gamma(E,V)$ through the potential $U(z,V)$ can be obtained by piece-wise linear approximation method. The $\rho(E,V)$ in the three-dimensional case is calculated as $\frac{m}{2\pi\hbar}\varepsilon(E)$, here the $\varepsilon(E)$ is unit step function. Σ($E,V$) accounts for the energies or speeds ranges of launched electrons, which can be expressed as:

$$\sigma\left( E,V \right)=\left\{ \begin{aligned} \varepsilon\left( E \right)-\varepsilon(E-eV), &eV<E_{F}, \\ \varepsilon\left( E \right)-\varepsilon(E-E_{F}), &eV\geq E_{F}, \end{aligned} \right.$$

Here, $E_{F}$ is the Femi energy-level of electrodes. In addition, the negative derivative of the Fermi function, described by $\frac{1}{4k_{B}T}{sech}^{2}(\frac{E}{2k_{B}T})$, is defined as the thermal broadening function. It represents the thermal variation in the speeds/energies of emitted electrons. Finally, the current in the three-dimensional tunneling device is deduced as [5]

$I=\frac{eS}{4\pi\hbar}\bigotimes\Gamma(E,V)\bigotimes\frac{m}{2\pi\hbar}\varepsilon(E)\bigotimes\sigma(E,V)\bigotimes\frac{1}{4k_{B}T}{sech}^{2}(\frac{E}{2k_{B}T})$,

where $S$ is the cross-sectional area.

The above parameters during the calculation were determined as:[6] $m_{eff, ITO}=0.44 m_{e}$, ${EF}_{ITO}=0.535 eV$, ${WF}_{ITO}=4.5 eV$, $m_{eff, Al2O3}=1.0 m_{e}$, ${EA}_{Al2O3}=2.3 eV$, $m_{eff, TiN}=1.0 m_{e}$, ${EF}_{TiN}=4.5 eV$, ${WF}_{TiN}=4.5 eV$, where $m_{eff}$, $EA$, $WF$ and $EF$ represent the electron effective mass, the electron affinity, the working function and the Fermi level respectively, the total tunneling current density of the three-dimensional mMQWs system is calculated as shown in the Figure S5.


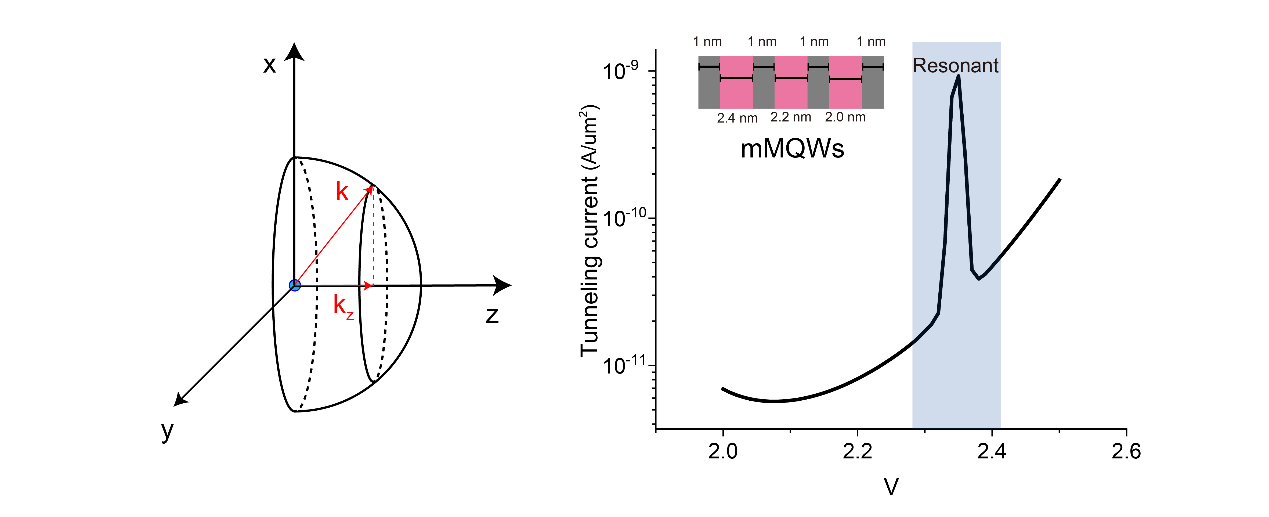
­

**Figure S5**. The calculated tunneling current density of the mMQWs structure.

**Section S8. An analogy between the resonant MQW and Fabry-Perot cavity**

As illustrated in Figure S6(a), a potential barrier can be likened to a gold mirror, where mirrors of varying thicknesses exhibit different reflectivities, representing distinct probabilities of electron transmission in the tunneling junction system. Similarly, only a photon at the resonant wavelength can attain maximum transmittance within the resonant cavity, just as resonant electron tunneling occurs when the electron energy matches the resonant energy level.

By introducing the concept of dielectric loss (k) in the Fabry-Perot cavity, we observe a decrease in photon oscillations and a subsequent decrease in the Q-factor, as depicted in Figure S6(b). We further analyze the resonance enhancement (the ratio of resonant transmittance to non-resonant transmittance, $\frac{T_{res}}{T_{non\_res}}$) in the Fabry-Perot cavity across different Q-factors (Figure S6(c)). When the thickness of the first mirror is decreased from 50 nm, the overall transmittance increases. However, optimal resonance conditions are only achieved with the highest Q-factor, where resonance enhancement effectively enhances the efficiency by increasing transmission. This resonant behavior shares similarities with electron resonance tunneling. Expanding on this analogy, the increasing number of MQWs (forming a resonant cavity) corresponds to an improvement in the Q-factor. It is only under high enough Q-factor conditions that ultra-strong resonance enhancement can effectively play the role in improving the tunneling rate, realizing both high photon-emission power and efficiency simultaneously as demonstrated in Figure 1(c).


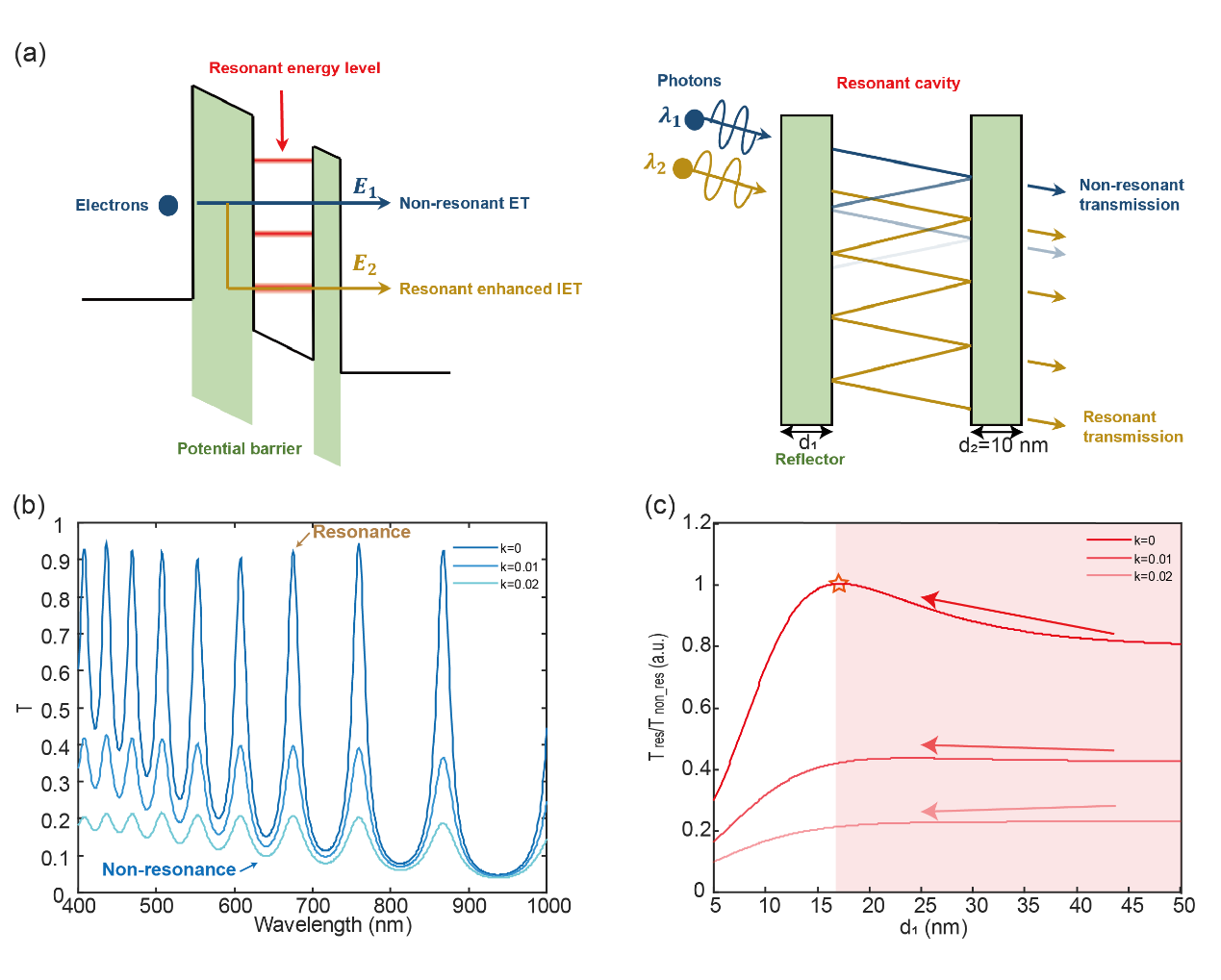
**Figure S6.** The physical explanation for working principle of mMQWs system. (a) The analogy between MQW system and Fabry-Perot cavity. (b) The transmission of multiple wavelengths in Fabry Perot cavity with different dielectric loss. Here, we use 10-nm gold mirrors on both sides to sandwich 1000-nm dielectric film (n=1.5) to form an optical resonant cavity. One can see the obvious Q-factor decrease with increasing the dielectric loss (k) from 0 to 0.02. (c) The resonance enhancement ($\frac{T_{res}}{T_{non\_res}})$ in different Q-factor optical cavity. Here, we use 626 nm as the non-resonant wavelength and 669 nm as the resonant wavelength. The optical transmittance is calculated based on the standard transfer matrix method (TMM) method.

**Section S9. Evaluation of the additional radiation loss introduced by the mMQWs system**

To evaluate the additional loss introduced by the multiple metallic quantum wells (mMQWs) system, we calculated the absorption of single metallic quantum well (MQW) and 3-MQWs system, as illustrated in Figure S7. Due to the ultrathin thickness of the metallic film, the results demonstrate that the 3-MQW system would introduce additional ~15-20% absorption loss within acceptable range.


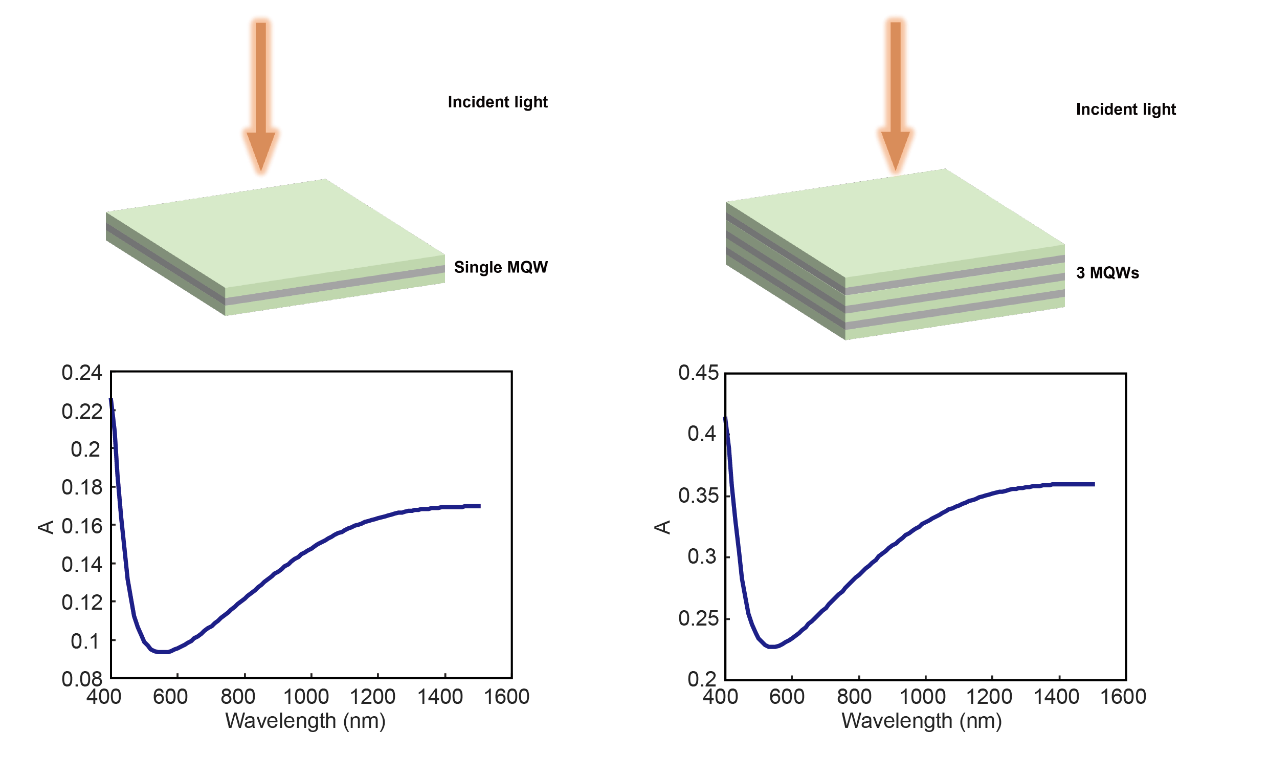


**Figure S7:** he radiation efficiency of single MQW and 3-MQWs system. Here, the MQW is constructed by 1-nm Al_2_O_3_/2-nm TiN/1-nm Al_2_O_3_. The material optical properties of Al_2_O_3_ and TiN are obtained from previous literatures [6-8].

**Section S10. The deviation of multiple RIET-enhancement factor caused by the fabricated uncertainty**

Considering the thickness uncertainty during the practical multilayers fabrication, we re-simulate the $EV\Gamma$ map with the quantum well thickness deviation of 0.2 nm (around an atomic layer) as shown in the inset of Figure S8. One can see from the $E$-$\Gamma$ curve at the 2.3 V bias voltage as shown in the Figure S8, the resonance enhancement factor is reduced as ${<10}^{7}$ fold due to the shift of resonant energy level. But it is still much higher than that of single MQW system (${10}^{4}$).


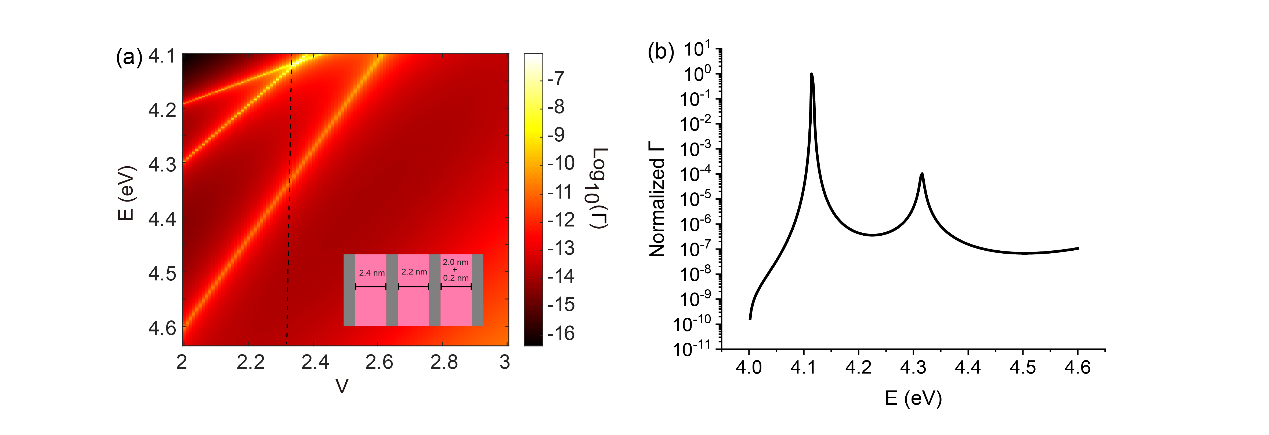


**Figure S8**. The deviation of RIET-enhancement factor with quantum well thickness deviation of 0.2 nm. **(**a) $EV\Gamma$ map of mMQWs system with one potential well thickness being increased by 0.2 nm. (b) The corresponding $E$-$\Gamma$ curve at 2.3 V bias voltage.

**References**

[1] H. Qian et al., "Highly-efficient electrically-driven localized surface plasmon source enabled by resonant inelastic electron tunneling," *Nat. Commun.,* vol. 12, p. 3111, 2021.

[2] M. Parzefall, and L. Novotny, "Light at the End of the Tunnel," *ACS Photonics,* vol. 5, pp. 4195-4202, 2018.

[3] K. F. Brennan, and C. J. Summers, "Theory of resonant tunneling in a variably spaced multiquantum well structure: An Airy function approach," *J. Appl. Phys.,* vol. 61, pp. 614-623, 1987.

[4] D. Y. K. Ko, and J. C. Inkson, "Matrix method for tunneling in heterostructures: Resonant tunneling in multilayer systems," *Phys. Rev. B,* vol. 38, pp. 9945-9951, 1988.

[5] P. F. Bagwell et al., "Resonant tunneling diodes and transistors with a one‐, two‐, or three‐dimensional electron emitter," *J. Appl. Phys.,* vol. 68, pp. 4634-4646, 1990.

[6] H. Qian et al., "Large optical nonlinearity enabled by coupled metallic quantum wells," *Light Sci. Appl.,* vol. 8, p. 13, 2019.

[7] H. Qian et al., "Nanoscale optical pulse limiter enabled by refractory metallic quantum wells," *Science Advances,* vol. 6, p. eaay3456.

[8] T. A. F. König et al., "Electrically Tunable Plasmonic Behavior of Nanocube–Polymer Nanomaterials Induced by a Redox-Active Electrochromic Polymer," *ACS Nano,* vol. 8, pp. 6182-6192, 2014.
